# Development of Advanced Aluminum and Magnesium Alloys: Microstructure, Mechanical Properties and Processing

**DOI:** 10.3390/ma17194811

**Published:** 2024-09-30

**Authors:** Wenming Jiang, Qingqing Li

**Affiliations:** State Key Laboratory of Materials Processing and Die & Mould Technology, School of Materials Science and Engineering, Huazhong University of Science and Technology, Wuhan 430074, China; liqq@hust.edu.cn

## 1. Introduction and Scope

Mg and Al alloys are the first and second engineering light metals, which are widely used in the aviation, aerospace, navigation, automotive, and electronic fields. With the rapid development of these fields, the demand to understand the properties of Mg and Al alloys is further increasing, and the requirements for large complex light Mg and Al alloys components become higher and higher. Therefore, high-performance advanced Mg and Al light alloys will have great application potentials in the future, which has also become a research hotspot. The development of advanced Mg and Al alloys involves in-depth studies of the microstructures, mechanical properties, and processing techniques of the Mg and Al alloys.

This Special Issue, entitled “Development of Advanced Aluminum and Magnesium Alloys: Microstructure, Mechanical Properties and Processing”, focuses on the latest progress in the innovative development of advanced aluminum alloys, magnesium alloys, and their composites. Particular attention has been paid to the relationships among the microstructure, mechanical properties, and processing conditions.

## 2. Contributions

This Special Issue contains a total of thirteen articles, with six papers focused on Al alloys, two papers on Mg alloys, and five papers on light metal composite materials.

### 2.1. Development of Advanced Aluminum Alloys

Kim et al. [1] investigated the optimization of 7xxx series aluminum alloys for low-pressure die-casting (LPDC) processes via simulation and experiments, to enhance propeller performance and durability. The increase in the Zn and Cu contents increased the hot-tearing susceptibility, while a sufficient Mg content of 2 wt.% mitigated this effect. Al-6Zn-2 g-0.5 u and Al-6Zn-2Mg-1.5Cu were identified as the optimized compositions. No hot tearing was observed in prototype castings of the Al-6Zn-2Mg-0.5Cu alloy.

Kim et al. [2] also developed a coupled thermal fluid stress analysis model using the ProCAST software to optimize the low-pressure die-casting (LPDC) of Al-6Zn-2Mg-1.5Cu alloy propellers. The optimal casting parameters such as the melt supply temperature, the initial mold temperature, and the curvature radius between the hub and blades were identified. The optimized propellers exhibited no significant internal defects and demonstrating improved structural integrity. The results highlight the practicality and efficiency of using simulation technology for product development.

Wang et al. [3] studied the effect of the torch angle on the formation accuracy, droplet transition behavior, and mechanical properties of a ZL205A aluminum alloy fabricated using the wire arc additive manufacturing process. The formation accuracy on the sidewall surface was increased when the torch angle increased to 100–120°. The obtuse torch angle was beneficial for small-size droplet formation and high frequency transition, contributing to improved deposition stability. At the obtuse torch angle of 100°, the pores were reduced, and the tensile strength and elongation of the depositions reached the maximum values of 258.6 MPa and 17.1%, respectively.

He et al. [4] investigated the high-temperature forming behaviors of a 7046-aluminum alloy through hot compression experiments. The flow stresses showed the dominant reducing characteristic with the increase in the compression temperature or the decrease in the strain rate. At a high strain rate or low compression temperature, the formation/interaction of substructures exhibited an intensified trend, while the extension of the dynamic recrystallization (DRX) grain boundaries was inhibited. Two constitutive models, involving a physically based (PB) model and a gate recurrent unit (GRU) model, were proposed to predict the hot compression features, and both models could accurately predict the hot compression behaviors of 7046-aluminum alloys.

Noga et al. [5] studied the microstructure and mechanical properties of Al-Si alloys produced using rapid solidification (RS) and hot extrusion. The Si particles of the Al-Si alloys produced using RS were close to spheroidal, with a comparable size. The Si particles as well as the Al-Si-Fe phase changed its shape from irregular to regular and spherical. The mechanical properties of the Al-Si alloys obtained by combining the RS and hot extrusion process were higher than that by gravity-cast and extruded materials. Compared to the gravity-cast and extruded alloys, they achieved increases of 20%, 25%, and 86% in the ultimate tensile strengths of AlSi5 RS, AlSi11RS, and AlSi20RS, respectively.

Arriaga-Benitez [6] reported the latest progress in creep-resistant aluminum alloys for diesel engine applications. The high-temperature stability of the aluminum casting alloys was determined by the formation of high-density uniform dispersoids with low solid solubility and low diffusivity in aluminum. The dispersion strengthening was an effective method to improve the creep deformation of Al-Si- or Al-Cu-based alloys. The future trends in developing heat-resistant aluminum alloys include their process optimization, alloy design, incorporation of additive manufacturing and aluminum-based matrix composites, and advanced coating technologies.

### 2.2. Development of Advanced Magnesium Alloys

Gubicza et al. [7] studied the annealing behavior of the Mg-0.9%Zn-2.05%Y-0.15%Al (at%) alloy, processed using rapidly solidified ribbon consolidation. The decrease in the amount of stacking faults due to recrystallization and the change in the chemical composition of the remaining solid material owing to the partial melting at 753 K and above decreased the ratio of the matrix lattice constants (*c*/*a*) of the alloy. The lattice constant change was reversible during heating and cooling. During annealing, both the matrix grains and the solute-enriched particles were coarsened. In addition, the changes to the morphology and the XRD intensity of the secondary phase were irreversible processes.

Chen et al. [8] studied the effects of bulk long-period stacking-ordered (LPSO) phases on the mechanical properties and fracture behavior of as-extruded Mg-Gd-Y-Zn-Zr alloys. The LPSO phases improved the tensile strength by refining the grain sizes. However, a small strengthening effect and a deterioration in the plasticity was provided by the bulk LPSO phases, as the compatible plastic deformation of the bulk LPSO phases was poor. Finally, a design strategy intended to maintain good solid-solution strengthening and fine-grain strengthening, optimizing the size and distribution of the bulk LPSO phase, was proposed for developing high-performance Mg-RE-Zn alloys.

### 2.3. Development of Advanced Light Metal Composite Materials

Zheng et al. [9] developed a dual-scale (nano and micron) particle-reinforced route to strengthen an Al 6061 alloy by introducing TiB_2_. The TiB_2_/6061 Al matrix composites were prepared using powder metallurgy. Metallurgical bonding between TiB_2_ particles and the A6061 matrix was achieved by two rounds of high-energy ball milling, and Al_3_Ti was synthesized in situ during sintering. The internal defects of the composites were eliminated during the hot-pressing process. The TiB_2_ particles were uniformly dispersed in the matrix. The hardness and tensile strength of the composites with a 5% mass fraction of TiB_2_ (1% micron + 4% nano) were 131 HV and 221 MPa, which were 79.5% and 93.9% higher than those of the pure matrix, respectively. In addition, the composites with a TiB_2_ content of 7% (3% micron + 4% nano) exhibited the optimal friction and wear properties.

Gutta et al. [10] developed a new high-strength and high-ductility aluminum metal–matrix composite. It was achieved by incorporating ceramic reinforcement into the metal, which was formed in situ from a polymer using pyrolysis. Firstly, the crosslinked PMHS polymer was introduced into pure aluminum via friction stir processing. Then, the micro- and nano-sized polymer was converted into ceramic particles by heating at 500 °C for 10 h and processed again via friction stir processing. The composite was strengthened due to the grain refinement and the larger ceramic particles, and it exhibited a 2.5- and 3.5-fold increase in yield strength and tensile strength, respectively, compared with the base metal.

Liu et al. [11] studied the interfacial structure and mechanical properties of AZ31B/carbon tool steel (SK7), AZ31B/carbon tool steel (DP980), and AZ31B/austenitic stainless steel (316L) fabricated via laser–gas tungsten arc welding and hybrid direct lap welding. The interface of the front reaction area and the keyhole reaction area of the AZ31B/SK7 and AZ31B/DP980 was mainly composed of an Fe-Al phase and Al-Mn phase. A multi-layer composite structure consisting of the Mg_17_Al_12_ layer and the Mg-Al eutectic layer was formed in the front reaction area of the AZ31B/316 joints. The tensile loads of the AZ31B/SK7, AZ31B/DP980, and AZ31B/316 joints were 283 N/mm, 285 N/mm, and 115 N/mm, respectively. The binding of Cr and Ni on Fe atoms at a lower reaction temperature promoted the formation of a brittle Mg-Al eutectic and Mg_17_Al_12_ and deteriorated the performance of the AZ31B/316 joints.

Kunčická et al. [12] developed a method to prepare Al/Cu laminated conductors, featuring two different stacking sequences using rotary swaging. The Al/Cu laminates had a diameter of 5 mm, with fine grains, more or less equiaxed. The maximum texture intensity of the Cu components of both the laminates was 2.3, indicating the grains had no dominant preferential texture orientations. The misorientations within the grains indicated that the residual stress was mainly presented in the Cu components. The laminates in both of the designed stacking sequences showed a comparable ultimate tensile strength of 280 MPa. However, the laminate consisting of an Al sheath and Cu wires showed lower plasticity due to the work hardening of Al.

Li et al. [13] developed an ultrasonic vibration-assisted compound casting method to prepare the Al/Mg bimetal composites. The results revealed that the ultrasonic vibration treatment (UVT) did not change the compositions of the interface. However, the phase distributions were improved. The coarse and gathered Mg_2_Si particles at the Al/Mg interface were refined and dispersed via the UVT. The shear strength of the Al/Mg bimetal composites with UVT was enhanced to 56.7 MPa from 33.3 MPa, which increased by 70.3%. This could be ascribed to the removal of the oxide film via the UVT, which improved the metallurgical bonding of the Al/Mg interface. In addition, the refined and uniformly distributed Mg_2_Si particles also led to the enhancement in the shear strength.

## 3. Outlook

This Special Issue of *Materials* attracted numerous submissions, and the final publication consists of 13 high-quality peer-reviewed articles. The studies presented here reflect the latest progress in the development of advanced aluminum and magnesium alloys and their composites. Deep insights into the relationships among the microstructure, mechanical properties, and processing were presented. We believe the knowledge shared will impact the future development of light-weight alloys and facilitate their industrial applications. As the Guest Editor, I would like to extend gratitude to all the authors, reviewers, and the editorial team for their contributions to this Special Issue.
